# Acute small-bowel toxicity during neoadjuvant combined radiochemotherapy in locally advanced rectal cancer: determination of optimal dose-volume cut-off value predicting grade 2–3 diarrhoea

**DOI:** 10.1186/s13014-015-0336-5

**Published:** 2015-01-31

**Authors:** Tina Reis, Edwin Khazzaka, Grit Welzel, Frederik Wenz, Ralf–Dieter Hofheinz, Sabine Mai

**Affiliations:** Department of Radiation Oncology, University Medical Center Mannheim, University of Heidelberg, Mannheim, Germany; Medical Clinic, University Medical Center Mannheim, University of Heidelberg, Heidelberg, Germany

**Keywords:** Locally advanced rectal cancer, Radiation enteritis, Dose-volume histogram, Dose-volume constraints, Neoadjuvant radiochemotherapy

## Abstract

**Background:**

Current therapeutic standard for locally advanced rectal cancer is the neoadjuvant radiochemotherapy with total mesorectal excision. Diarrhoea is the main acute side effect, induced by the dose to the small-bowel, frequently leading to a treatment modification. Aim of this study was to analyse the differences between the irradiated small-bowel volumes and the occurrence of acute diarrhea during combined radiochemotherapy for rectal cancer.

**Methods:**

45 patients treated with a neoadjuvant radiochemotherapy (three-field box 50.4 Gy; Cetuximab, Capecitabine, Irinotecan) for locally advanced rectal cancer within a prospective phase I/II study were evaluated. Based on the dose-volume histograms, the small-bowel volumes receiving doses of 5, 10 … 45 Gy (V5, V10 …V45) were calculated and compared with the prospectively documented small- bowel toxicities.

**Results:**

There was a statistically significant difference between irradiated small-bowel volumes and the severity of therapy related diarrhoea. The strongest validity concerning the risk of developing a grade 2–3 diarrhoea was seen at a dose level of 5 Gy (V 5) with a small-bowel volume of 291.94 cc. Patients with V 5 > 291.94 cc had significantly more often grade 2–3 diarrhoea, than patients with V5 below this cut-off value (82% vs. 29%; p < 0.0001).

**Conclusions:**

In the inverse treatment planning of rectal caner patients the small-bowel volume receiving 5 Gy should be limited to about 300 cc.

## Introduction

Since the CAO/AIO/ARO-94 trial [1,2] neoadjuvant radiochemotherapy (nRCT) followed by a total mesorectal excision (TME) is the standard treatment for locally advanced rectal cancer (LARC).

Five -fluoruracil (5-FU) or Capecitabine based chemotherapies concurrent to external beam radiotherapy (EBRT) are the most frequently used regimes. The last years new drugs such as Irinotecan, Oxaliplatin or Cetuximab combined with 5-FU/Capecitabine were introduced to improve treatment results.

Acute diarrhoea is one of the most common acute sequela of pelvic radiochemotherapy in up to 12-39% [3,4]. It often requires treatment and sometimes causes a therapy interruption resulting in a reduced efficacy. Although acute gastrointestinal toxicity is multifactorial [5], some studies described a statistical significant relationship between irradiated small-bowel volume and treatment induced diarrhoea during radiochemotherapy for rectal cancer [6,7]. Therefore an optimization of radiotherapy planning to spare small-bowel should be aspired. Data about which parameters of the dose-volume histogram (DVH) are to be optimized are rare [8,9].

Aim of this study was to analyse the differences between the irradiated small-bowel volume and the occurrence/severity of acute diarrhoea during combined radiochemotherapy with Capecitabine, Irinotecan and Cetuximab, using information extracted from the 3D treatment planning and the individual DVH from patients treated for LARC within a prospective study [10,11]. Moreover we identified cut-off values for the irradiated small-bowel volumes associated with diarrhoea grade 2–3. Additionally the difference between a high dose to a small-bowel volume and a little dose to a large small-bowel volume (“a lot to a little” or a “little to a lot”) will be discussed.

## Methods and materials

### Patients

45 patients treated with a nRCT (Cetuximab, Capecitabine, Irinotecan) for LARC between 2004 and 2007 were evaluated. All patients were treated in a prospective phase I/II study [10,11]. Median age was 60 years (range, 41–80 years).

Patient and tumour characteristics are shown in Table 1.

**Table 1:** **Patient and tumour characteristics**

**Characteristics**	**Number (n)**
Patients	45
Age, years	
Median	60
Range	41-80
Gender	
Male	37
Female	8
T category	
T1	0
T2	6
T3	36
T4	3
N category	
uN0	9
uN1	35
uN2	0
uNx	1
Metastases	
No	43
Liver	2
Surgery	
Anterior resection	36
Abdominoperineal resection	9

All studies on humans described in the present manuscript were carried out with the approval of the responsible ethics committee and in accordance with national law and the Helsinki Declaration of 1975 (in its current revised form), Informed consent was obtained from all patients included in studies.

### Treatment

All patients received weekly Cetuximab (400 mg/m^2^ day 1 and 250 mg/m^2^ days 8, 15, 22, and 29) 2 h before radiotherapy, weekly Irinotecan (40 mg/m^2^ days 1, 8, 15, 22, and 29) 1 h before radiotherapy, and Capecitabine orally twice daily (500 mg/m^2^ days 1–38).

Simulation and irradiation was done in prone position using a belly board. All patients received a 3D CT-based treatment plan. The abdomen and pelvis were scanned with 0.5-1.0 cm thick continuous slices.

The clinical target volume (CTV) included the primary tumour and the regional lymph nodes (mesorectal, presacral, internal/common iliac). The upper border of the CTV was at the L5–S1 interspace for cN0 and at L4–L5 for cN+ patients. The planning target volume (PTV) was defined as the CTV with 1 cm margins. The lower border of the PTV depends on the level of the primary tumour (rectal cancer > 6 cm from anocutaneous line: above the anal sphincter complex, provided a 3 cm margin from distal tumour edge; rectal cancer ≤ 6 cm from anocutaneous line: perineum). Organs at risk like the bladder or small bowel were contoured not exceeding L4. The small bowel was contoured as individual small bowel loops.

Radiotherapy was delivered with a linear accelerator using 18–23 MeV photons and a three-field box technique consisting of a posterior-anterior and 2 lateral fields. A total dose of 50.4 Gy was given in daily fractions of 1.8 Gy, 5 days a week. After a dose of 45 Gy, an additional dose of 5.4 Gy was given to the boost volume using a shrinking field technique based on treatment planning CT scan after oral contrast administration. The boost volume was defined as primary tumour including corresponding mesorectum with 2 cm margins circumferential and if possible 5 cm margins cranial.

### Toxicity scoring

Data were obtained retrospectively using the irradiation protocols and patient documents of the Department of Radiation Oncology and of the III. Medical Clinic, University Medical Centre Mannheim. Diarrhoea as a measure of acute small-bowel toxicity during nRCT was chosen as primary endpoint. Toxicity scoring was done prospectively during the whole radiochemotherapy session by use of questionnaires. The degree of diarrhoea was classified according to the NCI Common Toxicity Criteria (CTC) scale, version 3.0 (Table 2). In the analyses diarrhoea grade was used as dichotomized variable (grade 0–1 vs. 2–3). This classification was chosen because a diarrhoea grade ≥ 2 means an increase of 4 – 6 stools per day over baseline, which is a clinically important side effect, often leading to a therapy modification.

**Table 2:** **NCI Common toxicity criteria (CTC) scale, version 3.0 of diarrhoea**

**Diarrhoea grade 1**	**Grade 2**	**Grade 3**	**Grade 4**	**Grade 5**
Increase of <4 stools per day over baseline; mild increase in ostomy output	Increase of 4 – 6 stools per day over baseline; IV fluids indicated <24 hours; moderate increase in ostomy output compared to baseline; not interfering with ADL*	Increase of ≥7 stools per day over baseline; incontinence; IV fluids ≥24 hours; hospitalization; severe increase in ostomy output compared to baseline; interfering with ADL	Life-threatening consequences (e.g. hemodynamic collapse)	Death
Compared to baseline				

*Activities of Daily Living.

### Treatment plan analysis

For treatment planning small-bowel (from the Douglas pouch up to the L5-S1 region), large-bowel and other organs at risk were contoured based on the CT simulation scan with oral contrast.

From the extracted DVH of the PTV the small-bowel volumes that received doses of 5, 10, 15, 20, 25, 30, 35, 40 and 45 Gy (V5, V 10, …V45) were calculated. The determined small-bowel volumes were compared with the grade of the small-bowel toxicity. The volumes were only calculated in absolute numbers (cc) because the entire small-bowel was not contoured in all patients and therefore relative numbers would not be representative.

### Statistics

All analyses were performed using the IBM Statistical Package for Social Sciences software, version 19 (SPSS Inc., Chicago, IL). Data are presented as frequencies, medians, minimum and maximum values, averages and standard deviations. Spearman’s rank correlation coefficient was used. Pearson’s chi-squared test or rather Fisher’s exact test was used to assess changes in frequencies. Mann–Whitney *U*-Test or rather Student’s *t*-test was used to assess changes in averages.

Receiver operating characteristic (ROC) analyses were used to identify cut-off values for the irradiated small-bowel volumes associated with grade 2–3 diarrhoea. The area under the curve (AUC) and 95% confidence intervals (CIs) were determined for each ROC analysis. Cut-off values were identified using the Youden index, which is calculated as a linear combination of sensitivity and specificity (*Y = sensitivity + specificity-1*). The maximum of the Youden index indicates an optimal cut-off point [12]. A two-sided p value of < 0.05 was considered statistically significant.

## Results

### Clinical outcome

43 of the 45 patients received the planned total dose of 50.4 Gy. Two patients stopped radiotherapy early, one patient after a dose of 34.20 Gy because of a cholangitis, and one patient after a dose of 43.2 Gy of unknown reason.

**Figure 1:**
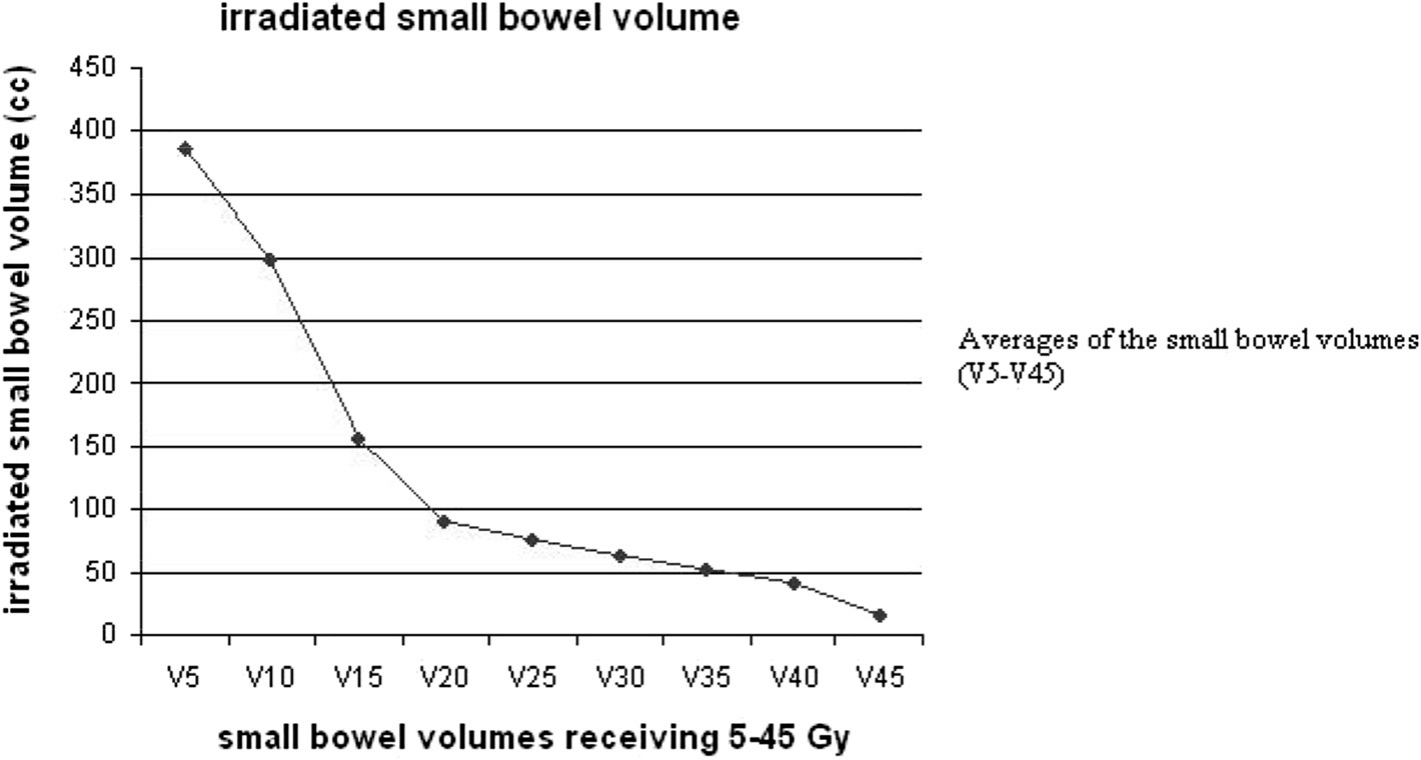
**Averages of the irradiated small bowel volume receiving 5 – 45 Gy (V5-V45 Gy).**

The average volume of the small-bowel was 585 cc (range, 121–983 cc; Figure 1). The average volume of the rectum was 1671 cc (range, 1061–2254 cc).

The median/average dose intensities (applied dose divided by the planned dose multiplied by 100 [%]) of Capecitabine, Irinotecan and Cetuximab were: Capecitabine 100/89 (range, 29–100); Irinotecan 100/93 (range, 33–100); Cetuximab 100/92 (range, 29–100).

20 patients received chemotherapy to 100%. In the remaining cases chemotherapy was interrupted and continued with reduced dose intensity due to different intolerances.

### Toxicity

Diarrhoea was the most frequent acute side effect. Overall 39 patients suffered from diarrhoea as follows: 6, 11, 15 and 13 patients had grade 0, 1, 2 and 3 respectively. Other acute side effects were: nausea (19 patients with grade 1 or 2, one patient with grade 3); abdominal pain (15 patients with grade 1 or 2, two patients with grade 3).

### Comparison of toxicity & determination of cut-off doses

**Figure 2:**
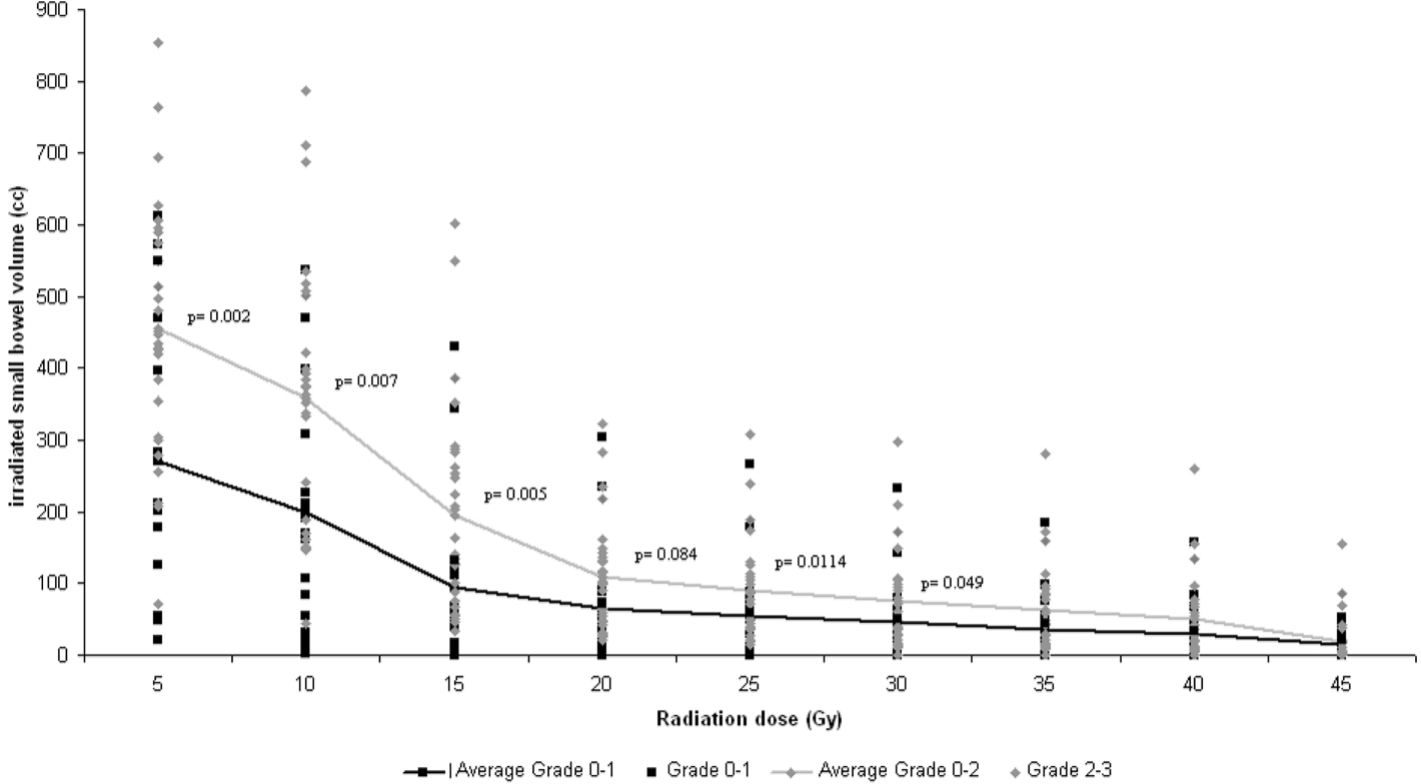
**Dose-volume histograms for small bowel.** Dose-volume histograms classified in diarrhoea grade 0–1 versus grade 2–3. Dots indicate small-bowel volume in individual patients receiving 5, 10…45 Gy. Lines indicate average volumes as a function of dose level.

17 patients with grade 0–1 versus 28 patients with grade 2–3 diarrhoea were compared. The average V5, V10, V15 and V30 was significantly larger in patients experiencing grade 2–3 diarrhoea compared to patients with grade 0–1 diarrhoea (p = 0.002, p = 0.007, p = 0.005 and p = 0.049; Figure 2).

ROC analyses were used to identify cut-off values predicting a diarrhoea grade 2–3. For the irradiated small-bowel volumes, which received a dose of 5 to 30 Gy, we had seen areas under the curve (AUC’s) between 0.68 und 0.77 (p < 0.05, Table 3). The highest AUC values were achieved for the V5 and the V15. The best cut-off point predicting a Grade 2–3 diarrhoea was seen for the V5 with a threshold-volume of the irradiated small-bowel of 291.94 cc (sensitivity 82%, specificity 71%, Youden-Index 0.53; Table 4). 23 of 28 patients (82%) with V 5 > 291.94 cc developed a grade 2–3 diarrhoea versus only 5 of 17 patients (29%) with V5 ≤ 291. 94 cc (p = 0.001).

**Table 3:** **ROC-analyses for determination of optimal thresholds for diarrhoea grade 2-3**

			**95% confidence**	**interval**
	**AUC**	**p**	**Lower confidence limit**	**Upper confidence limit**
V5	**0.768**	**0.003**	**0.616**	**0.920**
V10	**0.716**	**0.016**	**0.557**	**0.875**
V15	**0.752**	**0.005**	**0.597**	**0.907**
V20	**0.723**	**0.013**	**0.558**	**0.887**
V25	**0.708**	**0.020**	**0.541**	**0.875**
V30	**0.676**	**0.049**	**0.508**	**0.845**
V35	0.671	0.056	0.502	0.841
V40	0.651	0.092	0.481	0.822
V45	0.500	1.000	0.318	0.682

*Area Under the Curve.

**Table 4:** **Determination of optimal cut-off values for the irradiated small-bowel volume by varying radiation dose**

	Cut-off values (cc*)	**Sensitivity (%)**	**Specificity (%)**	**Youden**
V5	291.94	82.1	70.6	.53
V10	321.52	64.3	76.5	.41
V15	125,26	64.3	82.4	.47
V20	96,.84	53.6	88.2	.42
V25	13.50	100.0	41.2	.41
V30	9.62	96.4	41.2	.38
V35	7.03	96.4	41.2	.38
V40	4.85	89.3	41.2	.31
V45	1.24	67.9	47.1	.15

*cubic centimeter.

Moreover an irradiated small-bowel volume of 125.55 cc for a dose of 15 Gy could be determined as a second cut-off value. With a sensitivity and specificity of 64% and 82% this ratio is less significant and due to the low sensitivity for the clinical routine only restrictively applicable.

## Discussion

A significant difference between the irradiated small-bowel volumes and the severity of radiation induced diarrhoea were found, where the average volume of irradiated small-bowel was significantly larger in patients with grade 2–3 versus the volume in patients with grade 0–1 diarrhoea. Similar results were shown in recent studies. Gunnlaugsson et al. [6] found, that patients with diarrhoea grade 2+ had an average larger absolute volume of irradiated small-bowel for all cut-off doses. Baglan et al. [13] also described a statically larger volume of irradiated small-bowel at each 5 Gy level between 5–40 Gy in patients developing grade 3+ toxicity.

In the study of Gunnlaugsson et al. [6] 52% of the patients with an irradiated small-bowel volume of > 150 cc with >15 Gy showed a clinically significant diarrhoea in comparison to only 11% of the patients with >15 Gy in ≤150 cc of small-bowel. Similar results have been reported by Baglan et al. [13]. No patient with an irradiated small-bowel volume < 150 cc at a dose level of >15 Gy developed a grade 3+ diarrhoea versus 50% of the patients with a small-bowel volume of ≥150 cc.

In our study a classification in grade 0–1 versus grade 2–3 diarrhoea was chosen, because already grade 2+ diarrhoea may lead to a therapy modification and is therefore clinically relevant. We observed statistically significant higher grade 2–3 diarrhoea in patients with an irradiated small-bowel volume >291.94 cc in comparison to the patients with an irradiated small-bowel volume ≤291.94 at a dose level of 5 Gy. 82% of the patients with a small-bowel volume of >291.94 with 5 Gy had grade 2–3 diarrhoea, but only 29% of the patients with a small-bowel volume below this cut-off value. Therefore we suggest that the irradiated small-bowel volume receiving 5 Gy should be kept lower than 300 cc during treatment planning. However it must be stressed, that this analysis could be biased by the fact that small bowel, in the preoperative setting, may move during the treatment and thus a specific dose to a little part of the small bowel could be different at the end of the treatment than the dose calculated on the CT scan of treatment plan.

Overall therapy induced diarrhoea in our evaluation is unusually high. Overall 80% of the patients developed a diarrhoea during therapy, 28.9% of these patients a grade 3 diarrhoea. This could be caused by the additional application of systemic therapy, especially irinotecan and cetuximab. Neoadjuvant radiotherapy alone in patients with locally advanced rectal cancer has significantly lower overall toxicities compared to neoadjuavnt combined 5-FU based radiochemotherapy (2.9% vs. 14.9%, p < 0.001) [14]. Nevertheless, due to significant higher local control rates neoadjuvant radiochemotherapy followed by a total mesorectal excision (TME) is the standard treatment for locally advanced rectal cancer [15]. 5-FU must be administered as a continuous infusion during radiation, therefore the development of tolerable and efficient agents that do not require continuous infusion like oral fluoropyrimidine, e.g. capectiabine were promoted. The definitive demonstration that efficacy of concurrent radiochemotherapy with capecitabine is similar to 5-FU based radiochemotherapy has been provided by Hofheinz [16] and O’Connell et al. [17]. Reported grade 3+ diarrhea rates under capecitabine based radiochemotherapy were between 4%-24% [18-20]. In the phase III study of Hofheinz et al. overall 53% of the patients after capecitabine based radiochemotherapy suffer from diarrhea, whereas only 9% of the patients developed a grade 3+ diarrhea [16]. One approach to improve outcomes in rectal cancer is to deliver a second radiation sensitizing drug with effective systemic activity. Irinotecan is therefore good candidate. Compared to capecitabine alone combined radiochemotherapy with capectabine and irinotecan has higher overall diarrhea rates with similar grade 3+ diarrhea rates. In the study of Ugidos et al. [21] 64% of the patients after Irinotecan and capecitabine radiochemotherapy developed a therapy induced diarrhoea, 28% a grade 3+ diarrhea. In another phase II study an overall diarrhoe rate of 89% with a grade 3+ diarrhoea rate of 11% was reported [22]. The ongoing ARISTOTLE trial will provide a definitive answer about the benefit of adding irinotecan to capecitabine in the neoadjuvant setting [23].

Moreover a few studies showed an increased rate of side effects, especially accumulated toxicities of the small-bowel, during radiochemotherapy combined with Cetuximab [24,25], too. Nevertheless, although the absolute height of the observed side effects may be different after less toxic chemotherapy, the presented results can be extrapolated regarding thresholds and recommendations to standard chemotherapy. Furthermore, for rectal cancer acute effects on large bowel are difficult to distinguish from effects on small bowel.

For a long time it was controversially discussed if the dose distribution“ a lot to a little” or “a little to a lot”, which means a high dose to a small volume or a little dose to a large normal tissue volume is more critical for the induction of toxicity. This is especially relevant for intensity-modulated radiotherapy (IMRT), because IMRT typically delivers a low dose to a greater normal tissue volume than 3D conformal radiotherapy. So far this analysis was mainly done for the irradiation of lung cancer. Willner et al. [26] performed a systematic analysis of pneumonitis risk from DVH-parameters of the lung in patients with lung cancer treated with 3D conformal radiotherapy. Their data showed, that it is reasonable to disperse a low radiation dose (≤10 Gy) over a large volume if by this the high dose volume (>40 Gy) could be reduced.

In the treatment of pelvic tumours there are no clinically relevant data available up to now, discussing this item. Our data indicate in contrast to the situation for the lung a higher risk for diarrhoea, if large small-bowel volumes receive a low dose than other way round. Therefore we conclude” a lot to a little” is more favourable than “a little to a lot”. An illustrative presentation gives Figure 2 where the greatest differences between the two collectives with grade 0–1 and grade 2–3 diarrhoea, regarding the irradiated small-bowel volume were seen in the range of low radiation doses. Possibilities to reduce the volume of the irradiated small-bowel are prone positioning of the patient with a belly-board to achieve a small-bowel displacement away from the radiation field and application of highly conformal treatment approaches, such as intensity-modulated radiation therapy (IMRT). Several studies showed a significant reduction of dose to the small-bowel when using a belly board and therefore a significant decrease of irradiation induced enteritis [27-29]. IMRT has been applied to several pelvic malignancies, e. g. gynaecologic malignancy and anal cancer with reduced dose to the small-bowel and less toxicity compared to 3D conformal radiotherapy (3DCRT)[30-32].

In comparison, the data for IMRT in rectal cancer are relatively spare [33,34]. Mok et al. [34] made a pairwise comparison between IMRT and 3DCRT plans with respect to dose-volume histogram parameters. They found IMRT achieved a significant reduction of the mean dose and the absolute volumes of the small-bowel compared to the 3DCRT plans. Nevertheless it should be considered, that this fact applies to middle and high dose volumes and usually low dose volumes were equally or rather increased by IMRT compared to 3DCRT. Therefore it is all the more important to consider dose constraints during inverse treatment planning. According to our results the small-bowel volume receiving 5 Gy should be limited to about 300 cc.

## Conclusion

There is a significant difference between the small-bowel volumes and the severity of acute diarrhoea during nRCT for LARC. The highest significance for developing grade 2–3 diarrhoea was seen at a dose level of 5 Gy with a small-bowel volume of about 300 cc. This is clinically useful information and should be considered in future treatment planning processes where the small-bowel volume receiving 5 Gy should be limited to about 300 cc. Moreover we conclude, that “a little to a lot”, which means a little dose to a large small-bowel volume should be avoided to reduce the risk for radiation induced diarrhoea in contrast to the situation in the lung.

It is recommended that appropriate small-bowel dose–volume constraint using the currently available data should be introduced into routine inverse treatment planning for rectal radiotherapy.
